# The association between health literacy and self-management abilities in adults aged 75 and older, and its moderators

**DOI:** 10.1007/s11136-016-1298-2

**Published:** 2016-04-21

**Authors:** Bas Geboers, Andrea F. de Winter, Sophie L. W. Spoorenberg, Klaske Wynia, Sijmen A. Reijneveld

**Affiliations:** Department of Health Sciences, University Medical Center Groningen, University of Groningen, FA10, P.O. Box 196, 9700 AD Groningen, The Netherlands

**Keywords:** Health literacy, Older adults, Self-management, Well-being, Educational level

## Abstract

**Purpose:**

Low health literacy is an important predictor of poor health outcomes and well-being among older adults. A reason may be that low health literacy decreases older adults’ self-management abilities. We therefore assessed the association between health literacy and self-management abilities among adults aged 75 and older, and the impact of demographic factors, socioeconomic factors, and health status on this association.

**Methods:**

We used data of 1052 older adults, gathered for a previously conducted randomized controlled trial on Embrace, an integrated elderly care model. These data pertained to health literacy, self-management abilities, demographic background, socioeconomic situation, and health status. Health literacy was measured by the validated three-item Brief Health Literacy Screening instrument. Self-management abilities were measured by the validated Self-Management Ability Scale (SMAS-30).

**Results:**

After adjustment for confounders, self-management abilities were poorer in older adults with low health literacy (*β* = .34, *p* < .001). This was more pronounced in medium- to high-educated older adults than in low-educated older adults. Sex, age, living situation, income, presence of chronic illness, and mental health status did not moderate the association between health literacy and self-management abilities.

**Conclusions:**

Low health literacy is associated with poor self-management abilities in a wide range of older adults. Early recognition of low health literacy among adults of 75 years and older and interventions to improve health literacy might be very beneficial for older adults.

## Introduction

Health literacy is an increasingly important topic in public health. A large-scale health literacy survey in eight European countries estimates that around 47 % of European adults have low health literacy, i.e., that they have substantial problems with health-related tasks and situations [1]. Older adults are an especially vulnerable group with regard to health literacy [2–6]. Health literacy has been defined as ‘the degree to which people are able to access, understand, appraise, and communicate information to engage with the demands of different health contexts in order to promote and maintain good health across the life-course [7].’

Low health literacy has been shown to be an important predictor of various negative health outcomes, such as frequent hospitalization [8], higher mortality rates [9], and lower well-being [10]. The association between health literacy and well-being could be because low health literacy limits the self-management abilities of older adults. While many health literacy studies among older adults have focused on the association between health literacy and self-management behaviors in the healthcare context [11–15], the association between health literacy and self-management abilities (SMA) has largely been neglected. SMA consist of a general repertoire of cognitive and behavioral abilities for managing external resources in such a way that physical and social well-being is maintained or restored when lost [16]. SMA have been shown to be associated with major outcomes, such as well-being [17, 18] and health status [19].

However, evidence as to the association between health literacy and SMA is lacking, as is the case for its potential moderators. These moderators may include demographic factors like sex, age, and living situation. For example, living alone could indicate a lower level of social support; as a result, there could be too little social support to buffer the negative consequences of low health literacy [20]. Moreover, socioeconomic status, e.g., educational level and income, may also influence the association between health literacy and SMA. It could, for example, be possible for older adults with a higher educational level to have good self-management abilities, even if they have low health literacy.

This study therefore aims to (1) assess the association between health literacy and SMA among adults aged 75 and older, and (2) assess the impact of potential moderators (sex, age, living situation, educational level, income, presence of chronic illness, and mental health status) on this association.

## Methods

### Design and setting

This study consisted of secondary analyses of follow-up data from the stratified randomized controlled trial of Embrace, on adults aged 75 and older [21]. The study started in 2011 and was conducted in the eastern part of the province of Groningen, the Netherlands, which is one of the most deprived rural areas of the country. Participants were stratified into three risk profiles (i.e., robust, frail, and complex care needs), based on their level of frailty and their complexity of care needs. Next, balanced randomization was conducted per risk profile in order to achieve equal distributions across treatment groups of characteristics that could affect intervention outcomes [22]. The balancing criteria were sex, age, complexity of care needs, frailty, living situation, number of chronic conditions, whether or not receiving homecare, and whether or not receiving help with filling out the questionnaires. Embrace is a novel population-based elderly care model which aims to prolong the ability of older adults to age in place by meeting their needs through supporting integrated care [21]. The type and intensity of care and support that the participants received was based on their risk profile. Participants in the robust profile received low intensity care with a focus on self-management support and prevention; participants in the frail profile received high intensity care with a focus on psychosocial aspects; and participants in the profile with complex care needs received high intensity care with a focus on health care. The participants in the control group received care as usual, as provided by general practitioners and local health and community organizations. A more detailed description of the study design can be found in the published study protocol [21].

### Study population

Participants were recruited via their GPs and were eligible for inclusion if they were aged 75 years or older. Exclusion criteria were long-term stay in a nursing home, receiving an alternative type of integrated care, or participating in another research study. Of those eligible, 1456 older adults participated in the study (response rate 48.7 %). Non-respondents differed from respondents, with women, older participants, and participants from more rural areas more frequently declining to participate (all *p* values <.01).

A total of 1131 (78 %) participants responded to the follow-up questions. The most important reasons for loss to follow-up were refusal to continue participation (*n* = 106, 33 %), not filling out the questionnaires (*n* = 107, 33 %), moving to another city or a nursing home (*n* = 22, 7 %), and mortality (*n* = 75, 23 %). As expected in a population aged 75 years and older, dropouts (*n* = 325, 22 %) were significantly older, more frail, had more complex care needs, and had poorer health (all *p* values <.01). After exclusion of participants from residential care homes (*n* = 27, 2.4 %) and missing values for living situation (*n* = 10, .9 %), health literacy (*n* = 25, 2.2 %), and SMA (*n* = 17, 1.5 %), the responses of a total of 1052 participants were eligible for analysis in this study.

### Data collection

Written informed consent and baseline data were collected between October and December of 2011, and follow-up data were collected 12 months after the starting date [21]. Where needed, participants were assisted in filling out the questionnaires, either by family members, neighbors, etc., or by trained assistants. Data collection included measurements for health literacy, SMA, sex, age, living situation, educational level, income, presence of chronic illness, and mental health status. With the exception of educational level and sex, which were assessed at baseline, only data from the follow-up measurement were used for our current analyses.

The validated three-item Brief Health Literacy Screening (BHLS) was used to measure health literacy [23, 24]. The BHLS has been used in earlier studies on health literacy [25, 26]. The items of the BHLS are the following:

1. How often do you have someone help you read hospital materials?
2. How confident are you filling out medical forms by yourself?
3. How often do you have problems learning about your medical condition because of difficulty understanding written information?

These items were answered on a 5-point Likert scale (1–5). By reversing the scores on the second question and then summing up the scores of all three questions, a continuous total score (3–15) was calculated, with higher scores indicating higher levels of health literacy.

SMA were assessed with the validated Dutch version of the Self-Management Ability Scale (SMAS-30) [27, 28]. The SMAS-30 contains 30 items which are scored on 5- and 6-point Likert scales. The SMAS-30 consists of six subscales of five questions, each yielding scores in the range 0–100. Every subscale addresses one of the key domains of SMA (taking initiatives, be self-efficacious, investment behavior, positive frame of mind, multifunctionality of resources, and variety in resources) [28]. The total SMAS-30 score is based on the average of the six subscales, with a higher score indicating better SMA. If more than one item was missing for any of the subscales, no total score could be calculated for that subscale. In order to calculate the score for the full SMAS-30, scores for all six subscales had to be available. Examples of questions in the SMAS-30 are ‘Are you capable of taking good care of yourself?’ and ‘When things are not going so well, how often do you succeed in thinking positively?’

Potential moderators included sex, age, living situation, educational level, income, presence of chronic illness, and mental health status. Age was dichotomized as 75–80 versus >80 years. Living situation was dichotomized as living alone versus living with others (e.g., partner, siblings, or children). Educational level was dichotomized as medium to high (having finished at least secondary school) versus low. Income was dichotomized as high (over 1000 euro/month for people living alone or over 1350 euro/month for people living with others) versus low. The presence of one or more chronic illnesses was assessed by a single self-report question. Mental health status was assessed using the Mental Health Inventory (MHI-5), which is a subscale of a Dutch translation of the RAND-36 questionnaire [29]. This subscale consists of five questions that assess the participant’s feelings and emotions during the previous month. All five questions were rated on 6-point scales. Scores were averaged to a continuous total score if at least four of the questions were answered (range 0–100). Where fewer than four of the questions were answered, no total score could be calculated. There are multiple cutoff scores for the MHI-5. We adopted the commonly used cutoff point of 60 [30, 31], which led to one-quarter of our sample being classified as having a poor mental health status. To check whether this cutoff point influenced our results, we repeated the analyses with an alternative cutoff point (i.e., the median) and with the continuous scores. Both sets of analyses yielded results that were very similar to the primary analyses.

### Statistical analyses

We first explored the distribution of characteristics in the sample and their association with health literacy, using a series of independent samples *t* tests. Second, to assess the association between health literacy and SMA, we built a crude linear regression model with only health literacy as a predictor of SMA. Next, we added group (intervention/control), sex, and age to this model, to control for possible confounding effects. We then also assessed whether SMA were associated with an interaction between group (intervention/control) and Embrace risk profile; this was not the case. These analyses were then repeated for the various domains of SMA, in order to study the association between health literacy and SMA in more detail. Third, we assessed potential moderation of the association between health literacy and SMA by sex and age by separately adding to the model each variable and its interaction with health literacy, while adjusting for group (intervention/control). We then also assessed potential moderation by the other background characteristics by adding to the model each characteristic and its interaction with health literacy, while adjusting for group (intervention/control), sex, and age. If a characteristic showed a statistically significant interaction effect with health literacy, we repeated the analyses with this characteristic for the various domains of SMA. For all regression analyses, health literacy scores were centered around the mean to improve interpretability of the results [32].

To examine potential problems with multicollinearity in the regression analyses, variance inflation factors (VIFs) were calculated and examined for all regression analyses. Any VIF higher than 5 was considered to be an indicator of potential problems with multicollinearity. None of the VIFs reached this threshold (all VIFs <3.7).

All analyses were performed using SPSS 20.0 for Windows. We considered results to be statistically significant if *p* < .05.

## Results

The characteristics of the sample and the associations of these characteristics with health literacy are presented in Table 1. All studied characteristics were significantly associated with health literacy (all *p* values ≤.001). Health literacy was significantly lower for participants over 80 years of age, for women, for those with a lower educational level, for those with a lower monthly income, for those who lived alone, for those who suffered from chronic illness, and for those with poor mental health status.

**Table 1:** Characteristics of participants and corresponding levels of health literacy

	*n* (%)	Mean health literacy^a^ (SD)	*p*
Total	1052 (100)	11.70 (3.12)	
Sex			<.001
Male	468 (44.5)	12.28 (2.81)	
Female	584 (55.5)	11.24 (3.27)	
Age			<.001
75–80 years	470 (44.7)	12.49 (2.71)	
>80 years	582 (55.3)	11.07 (3.28)	
Living situation			<.001
With others	596 (56.7)	12.06 (2.90)	
Alone	456 (43.3)	11.24 (3.33)	
Educational level			<.001
Medium to high	526 (50.4)	12.61 (2.68)	
Low	517 (49.6)	10.75 (3.26)	
Income			.001
High	728 (85.2)	11.86 (3.08)	
Low	126 (14.8)	10.88 (3.23)	
Presence of chronic illness			<.001
No	380 (36.2)	12.27 (2.79)	
Yes	671 (63.8)	11.39 (3.25)	
Mental health status			<.001
Good	765 (72.9)	12.02 (2.94)	
Poor	285 (27.1)	10.85 (3.42)	

All *p* values based on independent samples *t* tests. Some data were missing for presence of chronic illness (.1 %), mental health status (.2 %), educational level (.9 %), and income (18.8 %). The higher percentage of missing data for income was the result of participants refusing to report their income (12.8 %) or not knowing their income (5.9 %)

^a^Range 3–15, with higher scores indicating higher health literacy

### Association between health literacy and SMA

Higher health literacy was significantly associated with better SMA (Table 2). Adjustment for group (intervention/control) did not change the strength of this association. Further adjustment for sex and age also had hardly any impact on the association.

**Table 2:** Association between health literacy and self-management abilities (*n* = 1052)

	*β* (95 % CI)	*p*
Crude model		
Health literacy	.34 (.29 to .40)	<.001
Model with intervention		
Health literacy	.34 (.29 to .40)	<.001
Intervention group	.01 (−.04 to .07)	.62
Model with sex and age		
Health literacy	.34 (.28 to .40)	<.001
Intervention group	.01 (−.04 to .07)	.65
Female	.08 (.03 to .14)	.005
Age over 80 years	−.06 (−.12 to −.01)	.031

The association between health literacy and the various domains of SMA, while being adjusted for group, age, and gender, are presented in Table 3. Health literacy was significantly and positively associated with all domains of SMA (all *p* values <.001).

**Table 3:** Associations between health literacy and the domains of self-management abilities (*n* = 1052)

Domain	*β* (95 % CI)	*p*
Taking initiative	.33 (.27 to .39)	<.001
Be self-efficacious	.30 (.24 to .36)	<.001
Investment behavior	.34 (.28 to .40)	<.001
Positive frame of mind	.27 (.21 to .33)	<.001
Multifunctionality of resources	.19 (.12 to .25)	<.001
Variety in resources	.23 (.17 to .29)	<.001

All analyses were adjusted for group (intervention/control), age, and sex

### Moderation of the association between health literacy and SMA

The results of the moderation analyses are presented in Table 4. In all models, the association between health literacy and SMA remained substantial (all *β*’s ≥.29, all *p* values <.001).

**Table 4:** Influence of demographic factors, socioeconomic factors, and health status on the association between health literacy and self-management abilities

	Model with main effects		Model with interaction	
	*β* (95 % CI)	*p*	*β* (95 % CI)	*p*
Demographic factors				
Sex^a^ (*n* = 1052)				
Health literacy	.36 (.30 to .42)	<.001	.40 (.31 to .50)	<.001
Female	.08 (.03 to .14)	.004	.09 (.03 to .14)	.003
Health literacy × Female	–	–	−.05 (−.15 to .04)	.26
Age^a^ (*n* = 1052)				
Health literacy	.33 (.27 to .39)	<.001	.39 (.29 to .48)	<.001
Age over 80 years	−.07 (−.12 to −.01)	.028	−.06 (−.12 to .00)	.043
Health literacy × Age over 80 years	–	–	−.07 (−.16 to .03)	.16
Living situation^b^ (*n* = 1052)				
Health literacy	.35 (.29 to .41)	<.001	.39 (.30 to .47)	<.001
Living alone	.08 (.02 to .14)	.012	.08 (.02 to .14)	.012
Health literacy × Living alone	–	–	−.05 (−.13 to .03)	.19
Socioeconomic factors				
Educational level^b^ (*n* = 1043)				
Health literacy	.35 (.28 to .41)	<.001	.42 (.33 to .52)	<.001
Low educational level	.02 (−.05 to .08)	.62	.02 (−.04 to .08)	.51
Health literacy × Low educational level	–	–	−.10 (−.19 to −.01)	.033
Income^b^ (*n* = 854)				
Health literacy	.36 (.30 to .43)	<.001	.35 (.28 to .42)	<.001
Low income	−.06 (−.12 to .00)	.049	−.06 (−.12 to .01)	.09
Health literacy × Low income	–	–	.04 (−.02 to .11)	.21
Health status				
Chronic illness^b^ (*n* = 1051)				
Health literacy	.33 (.27 to .39)	<.001	.31 (.21 to .42)	<.001
Presence of chronic illness	−.11 (−.16 to −.05)	<.001	−.11 (−.17 to −.05)	<.001
Health literacy × Presence of chronic illness	–	–	.02 (−.09 to .12)	.72
Mental health status^b^ (*n* = 1050)				
Health literacy	.32 (.26 to .37)	<.001	.29 (.22 to .36)	<.001
Poor mental health status	−.16 (−.21 to −.10)	<.001	−.15 (−.21 to −.10)	<.001
Health literacy × Poor mental health status	–	–	.05 (−.02 to .12)	.20

^a^Adjusted for the effects of group (intervention/control)

^b^Adjusted for the effects of group (intervention/control), age, and sex

#### Demographic factors

After adjusting for health literacy and intervention effects, we found that women showed significantly better SMA than men, but the interaction effect between health literacy and sex was not statistically significant. We found a similar pattern of results for age: Higher age was significantly associated with poorer SMA, but the interaction effect between health literacy and higher age did not reach significance. After additionally adjusting for both sex and age, we found that people who lived alone showed better SMA compared to people who lived with others, but there was no significant interaction with health literacy.

#### Socioeconomic factors

**Fig. 1:**
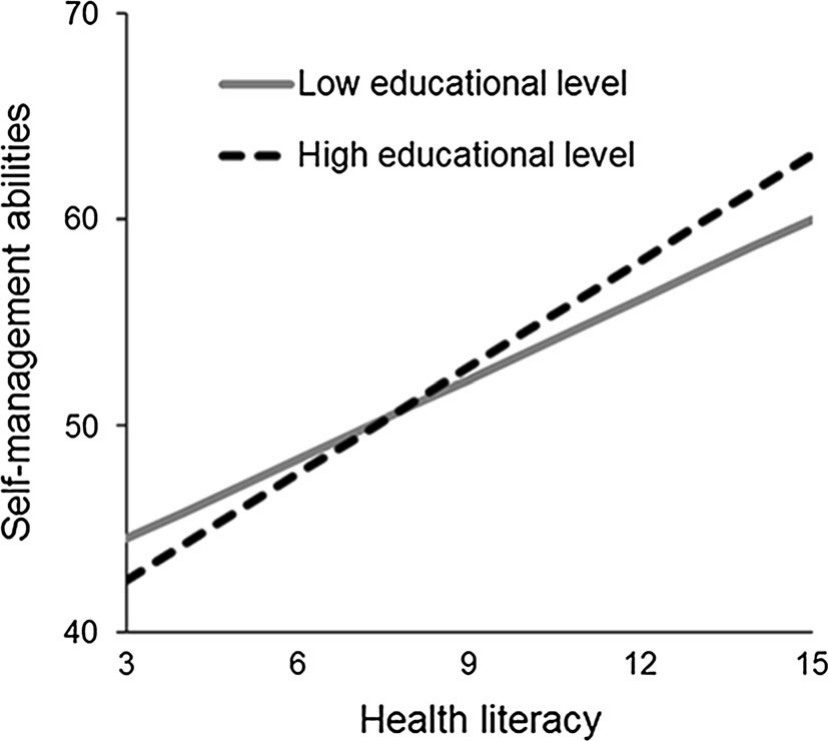
Association between health literacy and self-management abilities, split by educational level and adjusted for group (intervention/control), age, and sex

The models involving socioeconomic factors, i.e., education and income, show that lower educational level was not significantly associated with SMA after adjusting for health literacy and potential confounders. There was, however, a significant interaction effect between lower educational level and health literacy on SMA. The associations between health literacy and SMA, split by educational level, are presented graphically in Fig. 1. This figure shows that health literacy was a better predictor of SMA in people with medium-to-high levels of education, compared to those with lower levels of education. SMA were poorest in medium- to high-educated older adults with low health literacy. In total, 15.2 % (*n* = 83) of the participants with a medium-to-high educational level and 32.7 % (*n* = 180) of the participants with a low educational level had a health literacy score of 9 or lower. The analyses with educational level were repeated with the various domains of SMA as outcome variables. These analyses revealed that there was no statistically significant interaction effect between health literacy and educational level on any of the separate domains of the SMAS (all *p* values >.05, not tabulated). However, the interaction effect between health literacy and educational level approached significance for the domains of keeping a positive frame of mind (*β* = −.09, *p* = .058), taking initiatives (*β* = −.09, *p* = .064), and keeping a variety of resources (*β* = −.09, *p* = .078). Low income was significantly associated with poorer SMA, but its interaction effect with health literacy was not significant.

#### Health status

Finally, the results of the models with chronic illness and mental health status are also presented in Table 4. The presence of chronic illness was a significant predictor of poor SMA, but there was no significant interaction effect with health literacy. Poor mental health status was also significantly associated with poor SMA, but its interaction effect with health literacy did not reach significance.

## Discussion

This is the first study to examine the association between health literacy and self-management abilities (SMA) in community-dwelling adults of age 75 and older. Our results show that lower health literacy is associated with poorer SMA and all its separate domains, also after adjusting for potential confounders and moderators: group (intervention/control), sex, age, living situation, educational level, income, presence of chronic illness, and mental health status. The relationship between health literacy and SMA was moderated only by educational level.

No previous studies have assessed the association between health literacy and SMA and its domains among older adults. However, the various domains of SMA are known to be strongly associated with well-being [17, 18, 28], and well-being has also been shown to be associated with health literacy in a study among Japanese adults, most of whom were over 50 years of age [10]. Other partly comparable studies focused mainly on the associations between health literacy and self-management behaviors of older adults in the healthcare context. Some of these findings indicate an association of health literacy with poor glycemic control in patients with diabetes [12] and with poor self-management of asthma [13]. However, other studies found no association between health literacy and medication adherence among older adults [14, 33]. An explanation may be that older adults with low health literacy remain able to deal with specific self-management tasks, such as medication adherence [14, 33], but cannot perform tasks with multiple components, such as disease management [12, 13] or general self-management. Associations exist between health literacy and all domains of SMA, which suggests that health literacy is associated with SMA in various ways.

The association between health literacy and SMA in our study does not automatically imply a causal relation, as we used cross-sectional data. For example, low health literacy may lead to poorer health outcomes, which may lower a person’s SMA. Also, other factors may play a role in the association. An example of such a factor could be social networks, as people often draw on the health literacy skills of others in their social networks [34], and social networks are strongly associated with subjective well-being among older adults [35]. It is evident that the nature of the association between health literacy and SMA among older adults requires further study.

In our study, low health literacy was more strongly associated with low SMA in medium- to high-educated older adults than in low-educated older adults. No such association was found for any of the domains of SMA. In the previous studies, low health literacy was also found to be associated with low educational level [6, 36], but to our knowledge none of these studies reported that educational level moderates the impact of health literacy. We could not identify a specific domain that explains this association. The small group of older adults with medium-to-high educational level and low health literacy seems to be relatively vulnerable for poor SMA. One alternative explanation might be that low-educated older adults with both low health literacy and poor SMA are underrepresented in the current sample, as this group may be more likely to move to a care or nursing home before the age of 75. Given the relatively large number of analyses conducted in this study, this unexpected finding needs confirmation in future studies. Additionally, even though we found that SMA were poorest among medium- to high-educated older adults with low health literacy, low-educated older adults are still the more vulnerable group, as low health literacy is strongly associated with low educational level.

### Strengths and limitations

The main strength of our study was our use of a large community-based sample of adults aged 75 and older. Another strength of this study was the use of a broad and validated measure of SMA, specifically developed for older adults.

Some limitations of the current analyses should, however, be taken into account. First, the possibility of selection bias cannot be excluded as we used data from an intervention study with a response rate of 48.7 % and a follow-up rate of 78 %. However, we made use of a rather large community-based sample of adults aged 75 years and over, with few exclusion criteria, which is likely to improve the representativeness of the sample. We found some differences between responders and non-responders and between dropouts and participants in the follow-up measurement, as could be expected in a study among adults in this age group. Moreover, we studied associations (as opposed to prevalence rates) and we do not expect that the studied associations differ much between responders and non-responders. Second, even though data were longitudinally obtained, our analyses were cross-sectional in nature. This limited our ability to draw conclusions about the causality of the associations in the regression models. Third, as all data were collected by self-report, the possibility of information bias cannot be excluded.

### Implications

Professionals working with older adults should be aware of the association between health literacy and SMA in this group. This may also imply the need to include routine assessment of health literacy in assessment procedures for older adults. Such assessment can help professionals to identify the population at greatest risk for poor health outcomes and low well-being in the future. If low health literacy causes a decrease in SMA among older adults, future interventions should focus on mitigating the negative effects of low health literacy in this group. This could be done, for example, by assisting older adults to take the necessary steps to ensure their SMA on a longer term. Future longitudinal studies are needed to establish the causality of the studied associations.

Our results show that health literacy is associated with SMA and with all its domains among older adults. This association remains after adjusting for various combinations of factors, which suggests that this association exists in a wide range of older adults. As a result, many older adults with low health literacy may have a variety of problems stemming from poor SMA. For example, they may have low self-efficacy beliefs, fail to invest sufficiently in the future, and fail to keep a positive frame of mind. The potential moderating role of social factors in the association between health literacy and outcomes would be a relevant topic for future research. An example of this could be the quality of the relationship with the partner.

## Conclusions

We found that low health literacy is associated with poor SMA across a wide range of adults aged 75 and older. Additional studies are needed to establish the causality of the associations. Low health literacy was more strongly associated with poor SMA in people with a medium-to-high educational level, indicating that medium- to high-educated older adults with low health literacy are a vulnerable group for poor SMA. Early recognition of low health literacy among adults above the age of 75 could be very beneficial for this group.
